# Parathyroid Hormone-Mediated Hypercalcemia With Remarkable Bone Mineral Density Recovery After Multiple Zoledronic Acid Infusions

**DOI:** 10.7759/cureus.73435

**Published:** 2024-11-11

**Authors:** Meumbur P Kpughur-Tule, Carly M Hubers, Mahvish Renzu, Kendall Conway, Alexander M Satei, Wael Taha

**Affiliations:** 1 Internal Medicine, Trinity Health Oakland/Wayne State University, Pontiac, USA; 2 Internal Medicine, Wayne State University School of Medicine, Detroit, USA; 3 Diagnostic Radiology, Trinity Health Oakland/Wayne State University, Pontiac, USA; 4 Endocrinology, Division of Endocrinology, Wayne State University School of Medicine, Detroit, USA; 5 Endocrinology, Detroit Medical Center, Detroit, USA

**Keywords:** bisphosphonate use, bone mineral density, osteoporosis, pth-mediated hypercalcemia, zoledronic acid

## Abstract

This case report describes a remarkable improvement in bone mineral density (BMD) in a 95-year-old female with parathyroid hormone (PTH)-mediated hypercalcemia following treatment with zoledronic acid. Despite her complex medical history, including chronic kidney disease (CKD) and osteoporosis, the patient experienced a significant increase in bone density, particularly in the left femoral neck, while maintaining stable renal function. This case highlights the efficacy and safety of zoledronic acid in elderly patients with osteoporosis and hypercalcemia, emphasizing the importance of careful monitoring to prevent renal complications. These findings offer valuable insights into the use of bisphosphonates in older populations with additional comorbidities.

## Introduction

Hypercalcemia, defined as elevated calcium levels in the blood, affects about 1%-2% of the population and has various etiologies, including primary hyperparathyroidism, malignancy, and granulomatous diseases [1]. The regulation of calcium homeostasis is intricately linked to parathyroid hormone (PTH), which is secreted by the parathyroid glands. PTH plays a pivotal role in maintaining calcium balance by increasing bone resorption, enhancing intestinal absorption of calcium via its effect on vitamin D metabolism, and promoting renal calcium reabsorption. Dysregulation of this system can lead to hypercalcemia. It is crucial to identify the underlying cause of hypercalcemia, as each requires a different management approach. Primary hyperparathyroidism (PHPT) accounts for the majority of hypercalcemia cases in outpatient settings [2]​, while malignancy-related hypercalcemia is more prevalent in hospitalized patients [3]. PHPT typically leads to hypercalcemia through excessive secretion of PTH, which increases calcium release from bones, absorption from the gut, and reabsorption by the kidneys​ [2]. PTH-mediated hypercalcemia often presents a diagnostic challenge and can require comprehensive testing to distinguish it from other causes of elevated calcium.

Zoledronic acid, a potent bisphosphonate, is often employed in the management of hypercalcemia, especially when conventional interventions prove insufficient. It works by inhibiting osteoclast-mediated bone resorption, thereby reducing serum calcium levels and increasing bone mineral density (BMD). Its use in elderly patients, particularly those with chronic kidney disease (CKD), requires careful consideration due to potential nephrotoxicity and the need for close monitoring. This report discusses a case of PTH-mediated hypercalcemia in a 95-year-old female with significant improvement in BMD following treatment with zoledronic acid. This case highlights not only the challenges of diagnosing and managing PTH-mediated hypercalcemia but also demonstrates the therapeutic benefits and risks associated with bisphosphonate treatment in complex cases involving advanced age and CKD.

## Case presentation

The patient, a 95-year-old female, presented with recurrent episodes of hypercalcemia. Her symptoms included fatigue, generalized abdominal pain, constipation, and confusion. Her past medical history includes hypothyroidism, osteoporosis, CKD, and a history of remote breast cancer, which was treated with a lumpectomy and has been without recurrence or bone metastases. During her first endocrinology appointment, she reported no bone pain, fractures, or renal stones and had never used lithium, hydrochlorothiazide, or steroids. Surgeries included a total hip arthroplasty on the right side, eye surgery, and a large ventral hernia repair. Her current medications included cholecalciferol 1,000 units, levothyroxine 75 mcg, and magnesium oxide 320 mg. The magnesium oxide was indicated for maintenance of normal levels. The patient has never smoked or used tobacco, does not consume alcohol, and has no history of drug use; she occasionally consumes caffeine. She walks with a walker and has a supportive family, with her son often accompanying her to medical appointments. 

Her initial laboratory workup at age 93 revealed that the bone turnover marker (BTM) beta-C-terminal telopeptide (CTx), was 1221 pg/mL indicating active bone metabolism. Serum calcium was elevated at 14.3 mg/dL. An expected normal calcium range is 8.6-10.3 mg/dL, as such our patient was out of normal limits. She also had an intact PTH level of 19.4 pcg/mL, with normal limits being between 12 and 88 pcg/mL for PTH. Additional labs showed that the patient had an albumin of 3.3 g/dL, creatinine of 1.39 mg/dL, glomerular filtration rate (GFR) of 35, phosphorus of 2.0 mg/dL, and magnesium of 1.6 mg/dL. The spine radiograph revealed generalized bone demineralization and moderate degenerative changes without acute fractures. Furthermore, a parathyroid single-photon emission computed tomography (SPECT CT) showed no evidence of parathyroid adenoma. Differential diagnoses for her hypercalcemia included metastatic disease, sarcoidosis, lymphoma, and vitamin D toxicity, all of which were subsequently ruled out.

The diagnosis of PTH-mediated hypercalcemia was made based on comprehensive lab findings prior to the administration of zoledronic acid. The patient's initial serum calcium level was markedly elevated, with an albumin-adjusted corrected calcium of 12.94 mg/dL, confirming hypercalcemia. Concurrently, the PTH level of 19.4 pg/mL, while within the low-normal range, was not adequately suppressed as would be expected in cases of non-PTH-mediated hypercalcemia. This indicated a degree of PTH dependence. Additionally, the 25-hydroxy vitamin D level was elevated at 108 ng/mL, likely influenced by supplementation, while the 1,25-dihydroxy vitamin D level remained within normal limits. The patient’s phosphorus level was low-normal, an atypical finding in CKD, where hyperphosphatemia is more common. Magnesium levels were within acceptable ranges and did not impact the interpretation. Despite a negative SPECT scan for parathyroid adenoma, clinical complexity introduced by factors such as CKD, dehydration, and periods of immobility may have obscured typical findings. These data points collectively support a PTH-dependent mechanism underlying the patient’s hypercalcemia.

**Figure 1:**
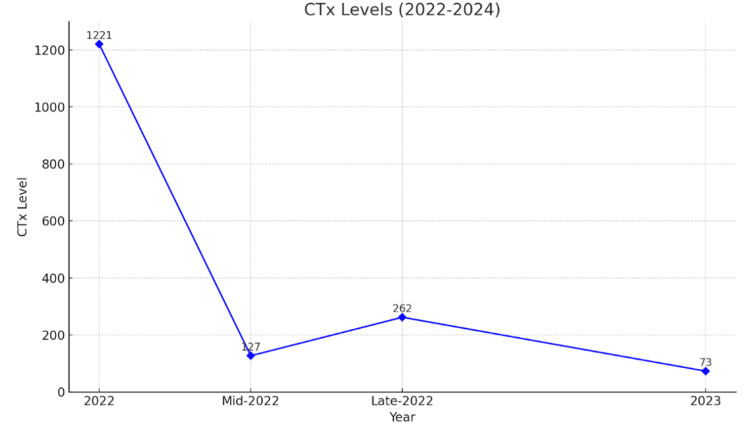
CTx levels CTx: Beta-C-terminal telopeptide CTx levels from 2022 to 2023, showing a significant decrease from 1221 pg/mL in early 2022 to 73 pg/mL in 2023, with a transient rise to 262 pg/mL in late 2022

**Figure 2:**
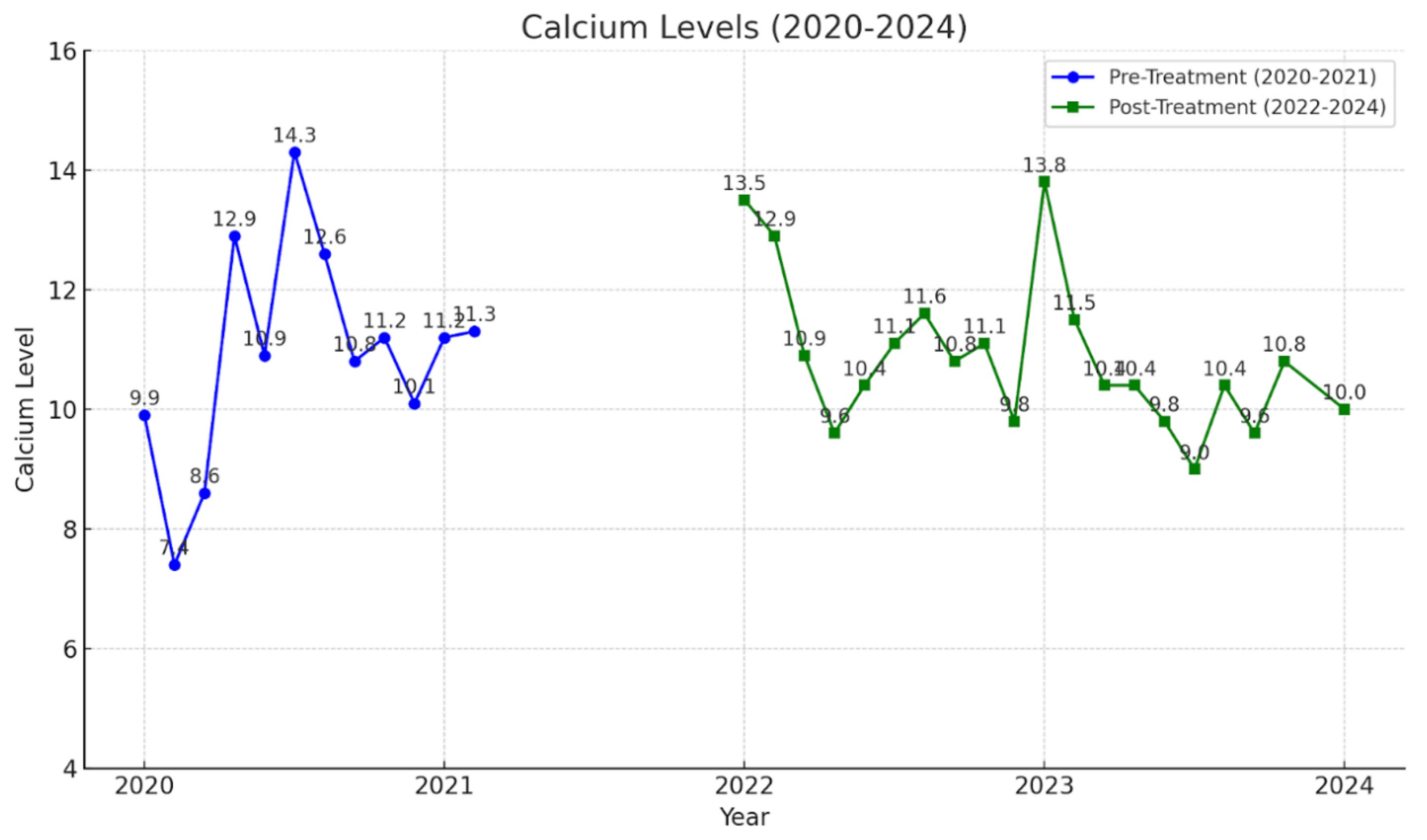
Calcium level trends Calcium level trends from 2020 to 2024, showing fluctuations pre- and posttreatment. The blue line displays calcium levels from 2020 to 2021, demonstrating initial fluctuations before treatment stabilization. The green line illustrates calcium levels from 2022 to 2024.  Both datasets provide comparative insights into calcium management over time

**Figure 3:**
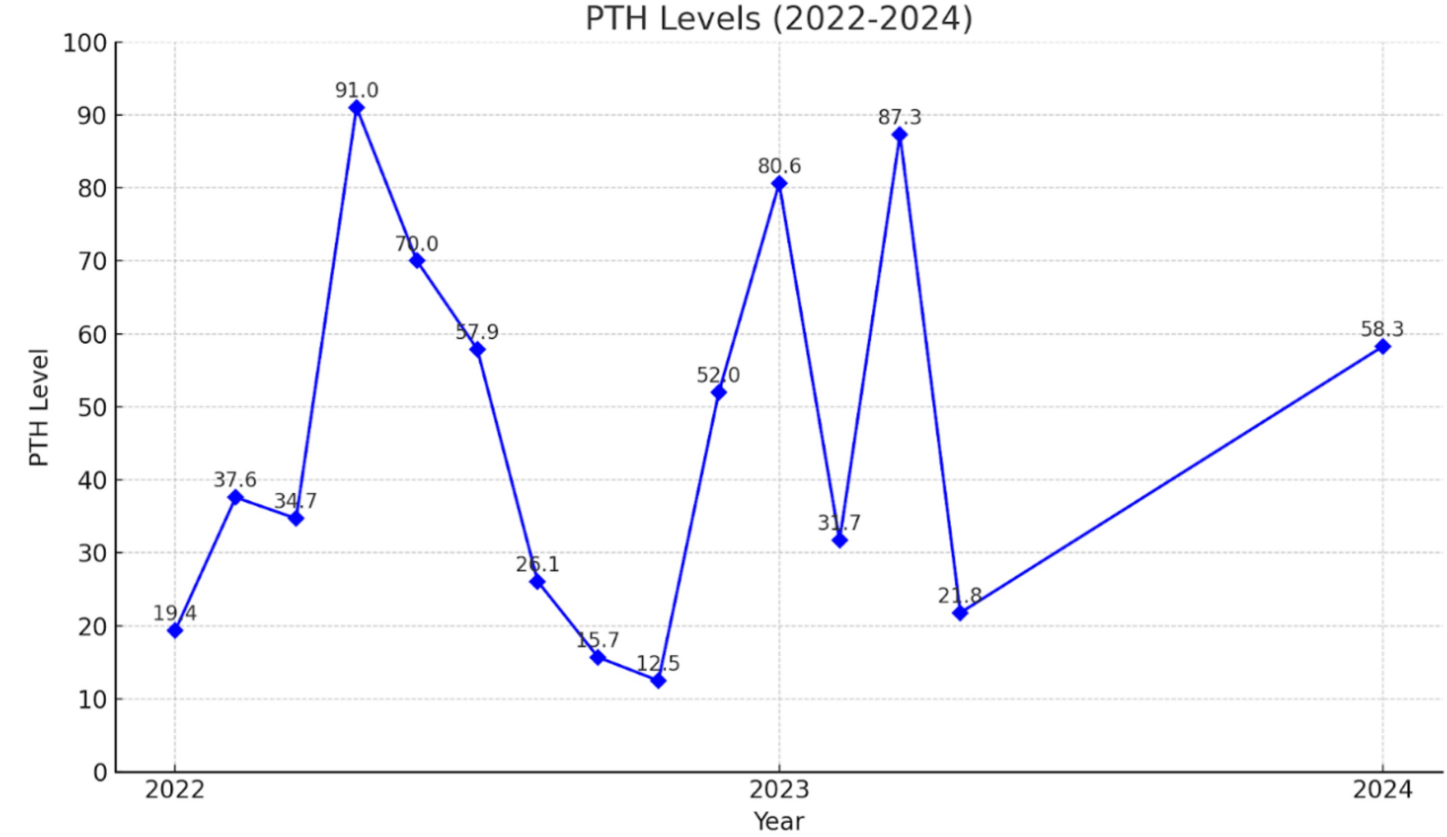
PTH levels PTH: Parathyroid hormone PTH levels from 2022 to 2024, showing notable fluctuations throughout the monitoring period. Levels peaked at 91 pg/mL in early 2023, followed by significant variability before rising again in 2024

To address these concerns, the patient received her first dose of intravenous (IV) zoledronic acid shortly after completing diagnostic studies. The patient was given 4 mg of zoledronic acid as a single IV infusion, administered over at least 15 minutes, on multiple occasions. Treatment frequency was based on the patient's clinical response. The IV zoledronic acid was used in combination with pamidronic acid as well to maximize results. The patient received multiple doses of IV zoledronic acid starting January 2021, with additional treatments on May 2023, August 2023, and May 2024. This therapy has significantly reduced beta-C-terminal telopeptide (a month later), serum calcium levels, and improvement in BMD over the following two years. Specifically, in the time following treatment initiation, the patient experienced a persistent reduction of CTx to 73 pg/mL (Figure 1) and a reduction of calcium to as low as 9 mg/dL (Figure 2); PTH levels peaked at 91 pg/mL and varied (Figure 3). As indicated by creatinine levels, renal function fluctuated but remained relatively stable. Phosphorus level remained mostly within normal limits.

**Figure 4:**
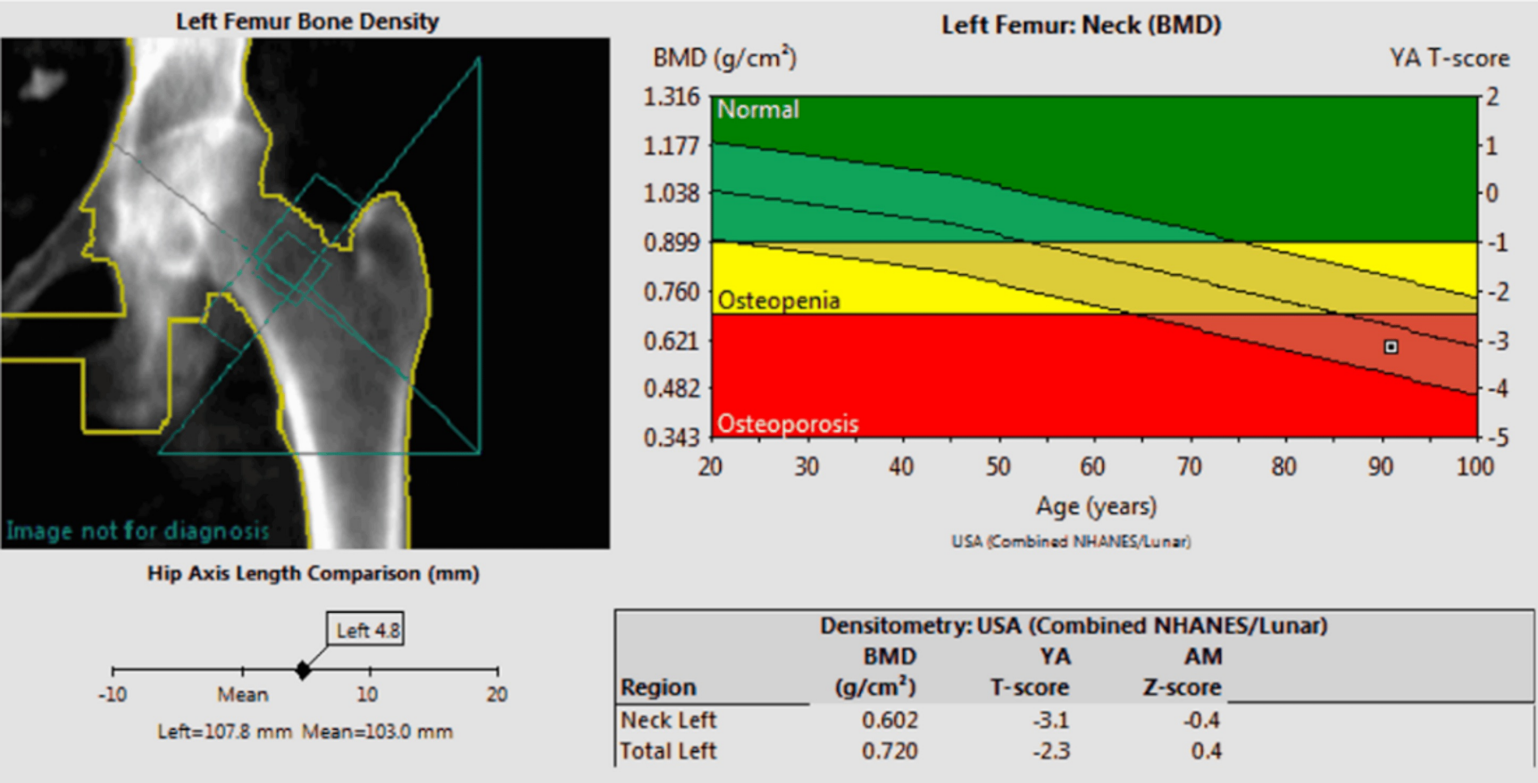
Pretreatment DEXA DEXA: Dual-energy X-ray absorptiometry; BMD: bone mineral density DEXA scan images obtained in 2020 of the left femoral neck reveals a BMD 0.602 and a T-score of -3.1

The patient's dual-energy X-ray absorptiometry (DEXA) revealed osteoporosis of the bilateral femoral necks and left forearm (Table 1). Before starting bisphosphonate therapy, her lumbar spine (L1-L4) BMD was 1.149 g/cm², with a T-score of -0.4 Her left femoral neck BMD was 0.602 g/cm², with a T-score of -3.1, consistent with osteoporosis (Figure 4). The left forearm BMD was 0.525 g/cm², with a T-score of -4.1, indicating severe osteoporosis.

**Table 1:** BMD values BMD: Bone mineral density BMD values, including T and Z-scores, from 2020 (pretreatment) to 2022 and 2024 (posttreatment). The WHO diagnostic criteria for T-scores are as follows: normal (>-1), osteopenia (between -1 and -2.5), and osteoporosis (≤-2.5)

Location:	Pretreatment T-score: 1/2020	Pretreatment Z-Score: 1/2020	Midtreatment T-score: 2/2022	Midtreatment Z-score: 2/2022	Posttreatment T-score: 4/2024	Posttreatment Z-score: 4/2024
Total AP lumbar spine	-0.4	1.8	0.3	1.9	-0.2	2.0
Lateral L2	-0.7	2.3	- 0.6	2.5	Restricted by lumbar scoliosis	Restricted by lumbar scoliosis
Lateral L3	-3.4	-0.4	-3.2	-0.2		
Lateral L4	-3.0	-0.4	-3.8	-1.1		
Left femoral neck	-3.1	-0.4	- 3.0	-0.1	-1.9	1.0
Right femoral neck	-2.8	-0.1	BMD not calculated due to fracture (arthroplasty)	BMD not calculated due to fracture (arthroplasty)	BMD not calculated due to fracture (arthroplasty)	BMD not calculated due to fracture (arthroplasty)
Left forearm	-4.1	-0.4	- 4.7	-0.8	-5.2	-1.2
Right forearm					-4.5	-0.4

**Figure 5:**
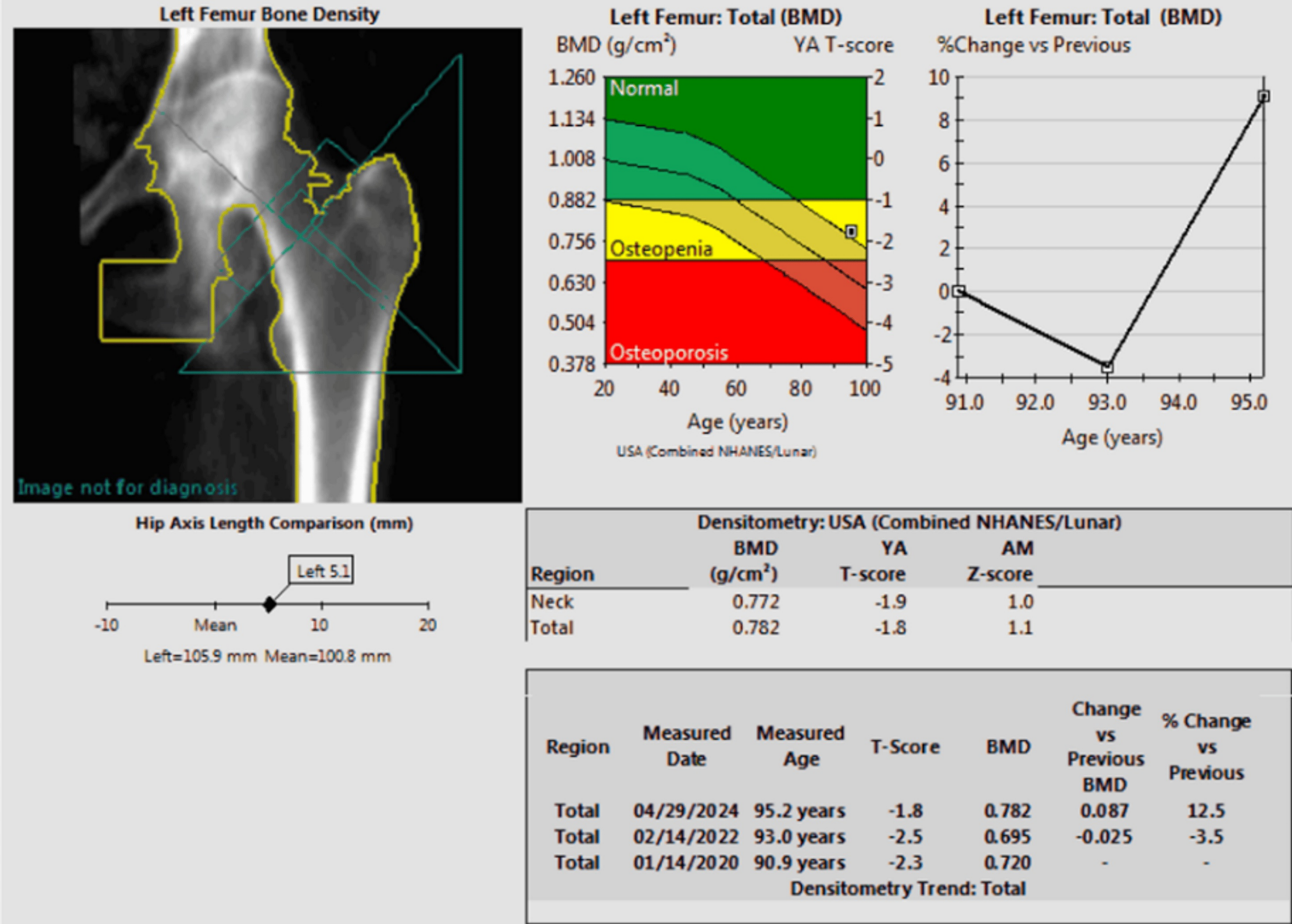
Posttreatment DEXA DEXA: Dual-energy X-ray absorptiometry; BMD: bone mineral density DEXA scan images obtained in 2024. Left femoral neck image reveals a BMD of 0.772 and a T-score of -1.9

After one year of treatment with zoledronic acid, the lumbar spine BMD slightly decreased to 1.156 g/cm², with a T-score of -0.3, showing stabilization of decline. The left femoral neck BMD showed a slight increase to 0.625 g/cm², with a T-score of -3, but the left forearm BMD decreased to 0.467 g/cm², with a T-score of -4.7. However, upon completion of two full years of zoledronic acid therapy, the lumbar spine BMD was 1.164 g/cm², with a T-score of -0.2. The left femoral neck BMD significantly improved to 0.772 g/cm², with a T-score of -1.9, reflecting a 12.5% increase (Figure 5).

Unfortunately, most recent scans did note that both forearms showed a decline in BMD, with the left forearm at 0.416 g/cm², with a T-score of -5.2, and the right forearm at 0.481 g/cm², with a T-score of -4.5, each with a -10.9% change, indicating a worsening score at this site. Overall, her response to zoledronic acid has been notably positive. It showed a trend toward stabilization in calcium levels and renal function with ongoing zoledronic acid therapy, despite her complex medical history, including CKD and osteoporosis. The patient tolerated the treatment with zoledronic acid well and did not report any significant adverse effects or side effects during the follow-up period.

## Discussion

Zoledronic acid is a potent bisphosphonate that acts primarily by inhibiting osteoclast-mediated bone resorption, which is critical in managing conditions like osteoporosis, hypercalcemia of malignancy, and Paget's disease of the bone. The mechanism of action involves its high affinity for hydroxyapatite crystals in the bone, where it binds and inhibits the enzyme farnesyl pyrophosphate synthase in the mevalonate pathway. This inhibition prevents the prenylation of small GTPase signaling proteins which are essential for osteoclast function and survival. As a result, osteoclast activity is reduced, leading to decreased bone resorption and turnover, increased BMD, and stabilization of calcium levels in the blood [4]. Zoledronic acid is particularly effective due to its long duration of action, allowing for infrequent dosing, which enhances patient compliance, especially in elderly patients like ours, and therapeutic outcomes.

Zoledronic acid typically leads to measurable improvements in BMD over time, particularly in the spine and hip, as seen in clinical trials like the HORIZON-PFT study, which demonstrated an increase in BMD of up to 6.7% in the lumbar spine and 5.06% in the femoral neck after three years of therapy in osteoporotic patients [5]. However, in our patient, the improvements in BMD, especially in the left femoral neck with a 12.5% increase, exceed the typical response observed in elderly populations, even in those receiving zoledronic acid.

While zoledronic acid is effective in treating bone-related conditions, it is associated with several risks, including renal impairment, especially in patients with pre-existing kidney conditions. As such, it is important to monitor renal function before and during treatment. Compared to other cases reported in the literature, the outcome of this patient reflects both expected benefits and notable deviations. Many elderly patients with osteoporosis and CKD are at higher risk for complications related to bisphosphonate use, particularly renal toxicity. However, our patient maintained stable renal function throughout the treatment period, highlighting that with appropriate monitoring, zoledronic acid can be safely used in patients with stage 3 CKD. Studies have shown that bisphosphonates can increase bone volume and mineralization without causing a dynamic bone disease in CKD patients, therefore making them a valuable option for managing osteoporosis in this population [6]. This supports the research suggesting that lower doses or extended intervals between doses may mitigate renal risks while maintaining efficacy [7].

Research on elderly populations often reports slower or more modest improvements in BMD due to age-related decreases in bone remodeling [8]. This makes our patient’s significant improvement even more remarkable and suggests that, despite her age and complex history, zoledronic acid was still able to significantly alter bone metabolism. The stabilization of her serum calcium levels also demonstrates its efficacy in addressing hypercalcemia, even when non-PTH mediated, another less commonly reported scenario. One possible explanation for the enhanced response could be the patient’s lack of other major risk factors for poor bone health, such as smoking or inactivity, which may have allowed for a more pronounced effect of the therapy. The absence of secondary contributors like these may have enabled a clearer, more direct impact of zoledronic acid on osteoclast inhibition and bone density preservation.

Similar to our patient, a case presented by Nurmi-Lüthje et al. highlights both the benefits and challenges of zoledronic acid treatment in elderly patients. While our patient experienced significant improvement in BMD, the patient in their report also saw positive effects, including long-term relief from chronic back pain after a single zoledronic acid infusion. However, both cases underscore the importance of monitoring for potential adverse effects like acute-phase reactions, which can be particularly severe, as seen in their patient who experienced muscle spasms and flulike symptoms. Although our patient did not encounter such extreme side effects, these cases emphasize the need for careful patient education and monitoring, especially in older individuals with complex medical histories [9].

## Conclusions

This case illustrates the successful management of PTH-mediated hypercalcemia in an elderly patient with complex medical comorbidities. The use of zoledronic acid significantly improved the patient's BMD and stabilized her calcium levels, demonstrating its efficacy and safety in this context. This case highlights the importance of a comprehensive diagnostic approach to hypercalcemia and the careful consideration of treatment options in patients with multiple comorbidities. Given the patient’s advanced age and CKD, her positive overall response to zoledronic acid offers valuable insights into the drug’s utility in PTH-mediated hypercalcemia and osteoporosis.
